# Physicochemical Evaluation of Insulin Complexes with QPDMAEMA-*b*-PLMA-*b*-POEGMA Cationic Amphiphlic Triblock Terpolymer Micelles

**DOI:** 10.3390/polym12020309

**Published:** 2020-02-03

**Authors:** Athanasios Skandalis, Anastasiia Murmiliuk, Miroslav Štěpánek, Stergios Pispas

**Affiliations:** 1 Theoretical and Physical Chemistry Institute, National Hellenic Research Foundation, 48 Vassileos Constantinou Avenue, 11635 Athens, Greece; thanos.skan@gmail.com; 2 Department of Physical and Macromolecular Chemistry, Faculty of Science, Charles University, Hlavova 2030, 128 40 Prague 2, Czech Republicmiroslav.stepanek@natur.cuni.cz (M.Š.)

**Keywords:** triblock terpolymers, polyelectrolyte/protein complexes, cationic polymers, insulin nanocarriers, protein nanocarriers

## Abstract

Herein, poly[quaternized 2-(dimethylamino)ethyl methacrylate-*b*-lauryl methacrylate-*b*-(oligo ethylene glycol)methacrylate] (QPDMAEMA-*b*-PLMA-*b*-POEGMA) cationic amphiphilic triblock terpolymers were used as vehicles for the complexation/encapsulation of insulin (INS). The terpolymers self-assemble in spherical micelles with PLMA cores and mixed QPDMAEMA/POEGMA coronas in aqueous solutions. The cationic micelles were complexed via electrostatic interactions with INS, which contains anionic charges at pH 7. The solutions were colloidally stable in all INS ratios used. Light-scattering techniques were used for investigation of the complexation ability and the size and surface charge of the terpolymer/INS complexes. The results showed that the size of the complexes increases as INS ratio increases, while at the same time the surface charge remains positive, indicating the formation of clusters of micelles/INS complexes in the solution. Fluorescence spectroscopy measurements revealed that the conformation of the protein is not affected after the complexation with the terpolymer micellar aggregates. It was observed that as the solution ionic strength increases, the size of the QPDMAEMA-*b*-PLMA-*b*-POEGMA/INS complexes initially decreases and then remains practically constant at higher ionic strength, indicating further aggregation of the complexes. atomic force microscopy (AFM) measurements showed the existence of both clusters and isolated nanoparticulate terpolymer/protein complexes.

## 1. Introduction

Diabetes mellitus is one of the most common and serious chronic diseases, affecting millions of patients worldwide [1,2]. The discovery of insulin was one of the most important scientific achievements of the last century [3]. Patients suffering from diabetes need to be administered with several doses of insulin daily, to maintain their blood glycose levels in the desirable rates. The main way for insulin administration is through subcutaneous injections [4].

Therefore, the need exists for the development of novel, effective nanocarriers that will employ alternative routes, such as oral administration, for the delivery of insulin. Presently, the evolution of synthetic polymer chemistry gives scientists the ability to design and synthesize polymers with tailored properties, a very important feature when it comes to gene/protein delivery applications [5,6]. Block polyelectrolytes are a very attractive class of polymers and they have been effectively used as nanocarriers for the delivery of genes and proteins, since they offer significant advantages, such as small size, good solubility and colloidal stability in aqueous solutions and high cellular uptake efficiency [7,8,9,10,11,12]. The complexation between polyelectrolytes and genes/proteins is mainly achieved via electrostatic interactions [13,14,15,16]. 

Cationic block polyelectrolytes have been widely used for the delivery of DNA [9,17] and proteins [18,19] since they carry positive charges that interact with the oppositely charged genes/proteins. Block polyelectrolyte design plays an important role in the structure and physicochemical/biological properties of the complexes formed. 

Polyelectrolytes offer certain advantages over other nanocarriers when it comes to insulin delivery applications, such as nanoscale size, protection from degradation and controlled release [2,18,20,21]. Moreover, such nanocarriers facilitate the uptake of insulin from routes other than invasive [2,4].

To the best of our knowledge, while several diblock copolymers and random copolymers (e.g., poly(ethylene glycol)-*b*-poly(l-lysine) (PEG-*b*-PLys) [22], and poly-lactic-*co*-glycolic acid, (PLGA) [23]), as well as ABA-type triblock copolymers (e.g., poly(ethylene oxide)-*b*-poly(ε-caprolactone)-*b*-poly(ethylene oxide) (PEO-*b*-PCL-*b*-PEO) and poly(2-(dimethylamino)ethyl methacrylate)-*b*-poly(ε-caprolactone)-*b*-poly(2-(dimethylamino)ethyl methacrylate) (PDMAEMA-*b*-PCL-*b*-PDMAEMA) polyelectrolyte complexes [20] and polylactic acid–*b*-polyethylene glycol–*b*-polylactic acid, (PLA-*b*-PEG-*b*-PLA) [24]) have been used as insulin delivery vehicles, there have not been reported any works employing micelles of ABC-type linear triblock terpolymers, most probably because the synthesis of such terpolymers is more laborious. 

In this work, QPDMAEMA-*b*-PLMA-*b*-POEGMA cationic triblock terpolymer micelles were used as nanocarriers for insulin (INS). The formation of the terpolymer/INS complexes was achieved through electrostatic interactions between the positive charges of the micelles and the negative charges on insulin. The complexation process was investigated, in a physicochemical aspect, by dynamic and electrophoretic light-scattering (DLS, ELS), atomic force microscopy (AFM) and fluorescence spectroscopy (FS). 

## 2. Materials and Methods

### 2.1. Materials

**Scheme 1:**
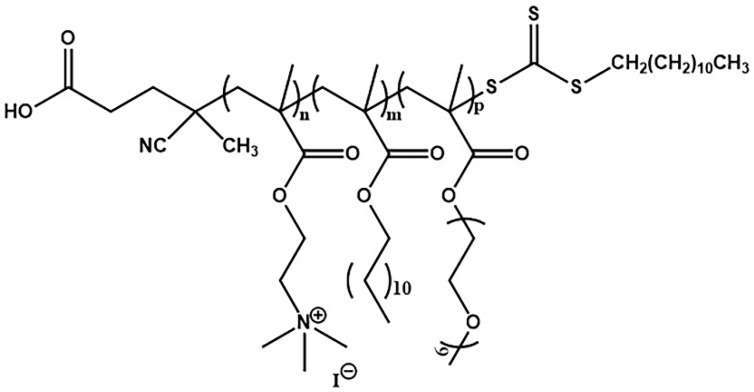
Chemical structure of QDPAEMA-*b*-PLMA-*b*-POEGMA triblock terpolymers.

Insulin (Mw = 5800 g·mol^−1^) was obtained from Sigma-Aldrich (Athens, Greece) and used without further purification. Dimethyl sulfoxide (DMSO, ≥99.9%, Sigma-Aldrich, Athens, Greece) and sodium chloride (NaCl, ≥99.0%, Sigma-Aldrich, Athens, Greece) were also used as received. QPDMAEMA-*b*-PLMA-*b*-POEGMA cationic triblock terpolymers were prepared in-house by quaternization of the tertiary amine groups of the PDMAEMA block of precursor PDMAEMA-b-PLMA-b-POEGMA triblock terpolymers, obtained by sequential reversible addition-fragmentation chain-transfer (RAFT) polymerization, using methyl iodide, (CH_3_I) as the quaternizing agent [25]. The chemical structure of the terpolymers is presented in Scheme 1.

### 2.2. Preparation of QPDMAEMA-b-PLMA-b-POEGMA/Insulin Solutions

QPDMAEMA-*b*-PLMA-*b*-POEGMA/INS complexes were formed by dropwise addition of INS solution (1 × 10^−2^ g·mL^−1^ in DMSO) to the terpolymer aqueous solution (5 × 10^−4^ g·mL^−1^) under gentle stirring in ambient conditions. The final volume of all mixed solutions was adjusted at 10 mL. The final concentration of the protein in the solutions ranged from 0.125–0.50 mg·mL^−1^.

### 2.3. Methods

Dynamic light-scattering measurements were conducted on an ALV/CGS-3 compact goniometer system (ALVGmbH, Hessen, Germany), equipped with an ALV 5000/EPP multi-τ digital correlator with 288 channels and an ALV/LSE-5003 light-scattering electronics unit for stepper motor drive and limit switch control. A JDS Uniphase 22 mW He-Ne laser (λ = 632.8 nm) was used as the light source. Measurements of the intensity correlation function were carried out five times for each concentration and angle and were averaged for each angle. The solutions were filtered through 0.45 µm hydrophilic PTFE filters (Millex-LCR from Millipore, Billerica, MA, USA) before measurements. The angular range for the measurements was 30–150°. Obtained correlation functions were analyzed by the cumulants method and the CONTIN software (ALVGmbH, Hessen, Germany). The size data and figures shown below are from measurements at 90°.

For the ionic strength dependent light-scattering measurements, the ionic strength of the polymer/INS solution increased by gradual addition of the appropriate volume of NaCl (using a 1 M stock solution). After each addition, the solution was rigorously stirred and left to equilibrate for 15 min before measurement. 

AFM measurements were performed in the semicontact (tapping) mode under ambient conditions using a NT-MDT NTEGRA Prima scanning probe microscope (NT-MDT Spectrum Instruments, Moscow, Russia) equipped with a Nanosensors silicon cantilever (Nanosensors, Neuchâtel, Switzerland). Aqueous polymeric solution (ca. 5 × 10^−4^ g/mL) were deposited on a freshly peeled out mica surface (flogopite, Geological Collection of Charles University in Prague, Czech Republic). The samples were dried in a vacuum oven at ambient temperature for 24 h. 

Fluorescence spectra were recorded on a Fluorolog-3 JobinYvon-Spex spectrofluorometer (model GL3–21, Kyoto, Japan). The excitation wavelength for the measurements was 280 nm.

Picosecond time-resolved fluorescence spectra were measured by the time-correlated single photon counting (TCSPC) method on a Nano-Log spectrofluorometer (Horiba JobinYvon, Kyoto, Japan), by using a laser diode as an excitation source (NanoLED, 375 nm) and a UV−vis detector TBX-PMT series (250−850 nm) by Horiba JobinYvon. Lifetimes were evaluated with the DAS6 Fluorescence-Decay Analysis Software (Kyoto, Japan).

## 3. Results and Discussion

The ability of QPDMAEMA-*b*-PLMA-*b*-POEGMA cationic triblock terpolymer micelles to complex with insulin through electrostatic interactions is investigated. The terpolymer/protein complexes were prepared in various INS concentrations, within the range C_INS_ = 0.125−0.5 mg·mL^−1^. The molecular characteristics of the terpolymers used are presented in Table 1.

**Scheme 2:**
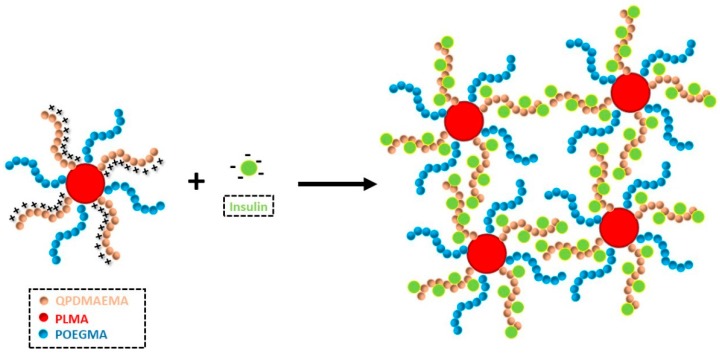
Schematic illustration of QPDMAEMA-*b*-PLMA-*b*-POEGMA triblock terpolymers micelles complexation with INS.

A schematic illustration of the formation of complexes from QPDMAEMA-*b*-PLMA-*b*-POEGMA triblock terpolymer micelles and insulin is presented in Scheme 2. Protein globules are expected to be complexed with the QPDMAEMA chains of the micellar corona and to occupy all available space within the corona. Some protein molecules located close to the periphery of the micelles act as bridges and enhance the formation of clusters of complexes. The neutral hydrophilic POEGMA chains in the corona contribute to the colloidal stabilization of the complexes/clusters.

Size and surface charge are very important parameters for the determination of the efficiency of polymeric systems in protein delivery applications. Thus, the complexes of QPDMAEMA-*b*-PLMA-*b*-POEGMA triblock terpolymers with insulin were investigated by light-scattering techniques (dynamic and electrophoretic) to gain information about their size and surface charge, respectively. It must be mentioned that all terpolymer/INS solutions prepared were colloidally stable and no precipitation phenomena were observed. This is a very important observation, especially for the complexes formed with QPDMAEMA_13_-*b*-PLMA_39_-*b*-POEGMA_8_ triblock terpolymer, which has the highest hydrophobic block (PLMA) ratio.

**Figure 1:**
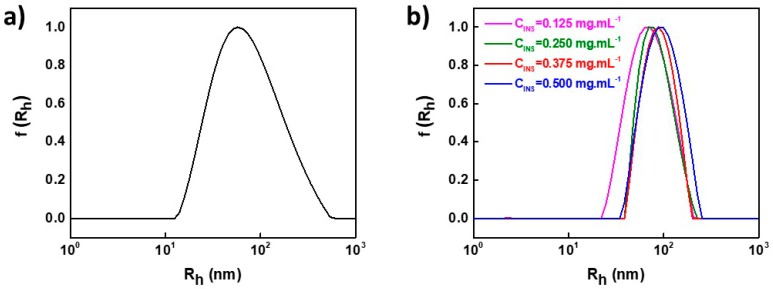
Size distribution graphs for (**a**) QPDMAEMA_13_-*b*-PLMA_39_-*b*-POEGMA_8_ micelles and (**b**) QPDMAEMA_13_-*b*-PLMA_39_-*b*-POEGMA_8_/INS complexes.

Figure 1a depicts size distribution graphs from DLS measurements (by CONTIN analysis) for QPDMAEMA_13_-*b*-PLMA_39_-*b*-POEGMA_8_ micellar aggregates. For comparison QPDMAEMA_13_-*b*-PLMA_39_-*b*-POEGMA_8_/INS complexes in all INS concentrations are shown in Figure 1b. Sizes of the complexes formed are larger than the initial terpolymer micelles indicating strong complexation with insulin. The complexes present monomodal size distributions in all cases. It is evident that there is a slight increase in the size of the complexes, as INS concentration increases, showing that the size of the complexes depends on the concentration of the protein in the solutions.

**Figure 2:**
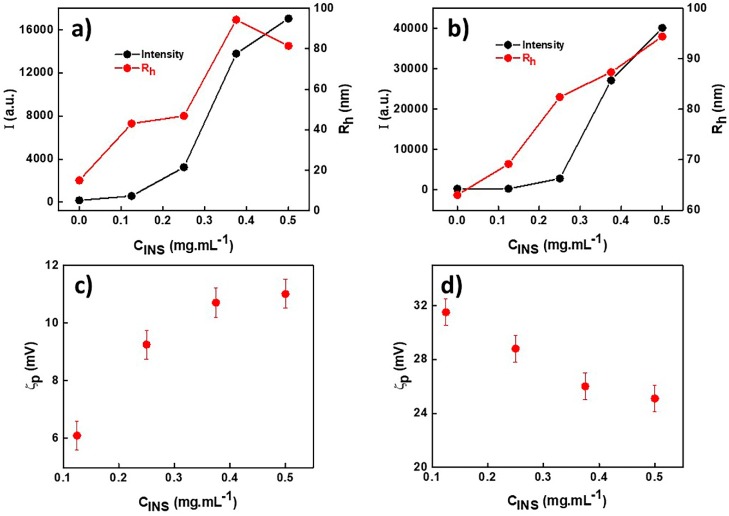
Variations in scattering intensity, I, R_h_ (**a**,**b**) and ζ-potential (**c**,**d**) as a function of C_INS_ for QDPMAEMA_33_-*b*-PLMA_16_-*b*-POEGMA_30_/INS (**a**,**c**) and QDPMAEMA_13_-*b*-PLMA_39_-*b*-POEGMA_8_/INS (**b**,**d**) complexes.

It was observed that both the scattering intensity and R_h_ of the complexes increase as INS concentration increases, for both terpolymers used (Figure 2a,b). More specifically, for QPDMAEMA_33_-*b*-PLMA_16_-*b*-POEGMA_30_/INS complexes (Figure 2a), the scattering intensity does not present significant changes at C_INS_ = 0.125 mg·mL^−1^, increases for C_INS_ = 0.25 mg·mL^−1^ and the greatest change is observed at C_INS_ = 0.375 mg·mL^−1^, where the scattering intensity increases approximately three times, showing the existence of particles with substantially larger mass. This is a possible indication for the formation of clusters of terpolymer/INS complexes in the solution, as the protein concentration increases and especially at higher protein concentrations. An increase in the scattering intensity is observed at C_INS_ = 0.5 mg·mL^−1^ as well. The hydrodynamic radius follows similar pattern and increases as INS concentration in the solution increases. At lower INS concentrations, the R_h_ value is ~45 nm and increases rapidly ~95 nm at C_INS_ = 0.375 mg·mL^−1^. At C_INS_ = 0.5 mg·mL^−1^ the R_h_ value is ~80 nm. Additionally, the fact that the size of the particles is larger than the size of the polymeric micelles before the complexation with insulin is a proof for the successful formation of the terpolymer/INS complexes.

Similar trends are observed for the QPDMAEMA_13_-*b*-PLMA_39_-*b*-POEGMA_8_/INS system (Figure 2b), with the only difference being that the scattering intensity values are much higher supporting the formation of clusters of complexes of higher mass. Moreover, the size of the complexes at low INS concentration does not show significant changes, compared to the size of the polymeric micelles before the interaction with insulin. This can be attributed to the lower QPDMAEMA block ratio and by extension, the lowest number of positive charges in the micelles of this particular terpolymer, which facilitates the formation of more compact clusters of complexes compared to the previous case.

The surface charge of all terpolymer/INS complexes solutions prepared was also investigated. It can be seen that the zeta potential (ζ_p_) values for QPDMAEMA_33_-*b*-PLMA_16_-*b*-POEGMA_30_/INS complexes are positive (Figure 2c), a result that confirms the scenario about the formation of clusters of complexes, as discussed earlier, which also implies that the periphery of the complexes (or clusters of complexes) are populated by terpolymer micelles and in particular cationic segments, mostly hiding the protein molecules in the interior of the clusters. For this to happen, each molecule of insulin must interact with more than one polymeric micelle forming bridges between terpolymer micelles. Furthermore, the ζ_p_ values become more positive (without large alterations) as INS concentration increases, indicating that the highest the INS concentration the highest the tendency for the formation of clusters of complexes. 

In the case of QPDMAEMA_13_-*b*-PLMA_39_-*b*-POEGMA_8_/INS complexes, ζ_p_ values are more positive and show a small decrease as INS concentration in the solution increases (Figure 2d). This probably means that a higher number of cationic segments populate the periphery of the clusters in this case and there are some subtle differences in the morphology of the clusters compared to the previous case. This behavior may be due to the QPDMAEMA block length being shorter in the QPDMAEMA_13_-*b*-PLMA_39_-*b*-POEGMA_8_ terpolymer.

According to literature, an increase in the ionic strength of the solutions of polyelectrolyte complexes with peptides or proteins can lead either to complex dissociation, since the electrostatic interactions between the components become weaker as a result of screening effects, or to secondary aggregation, or to precipitation of the original complexes due to lowering of solvent quality for the dispersed particles [26,27,28].

**Figure 3:**
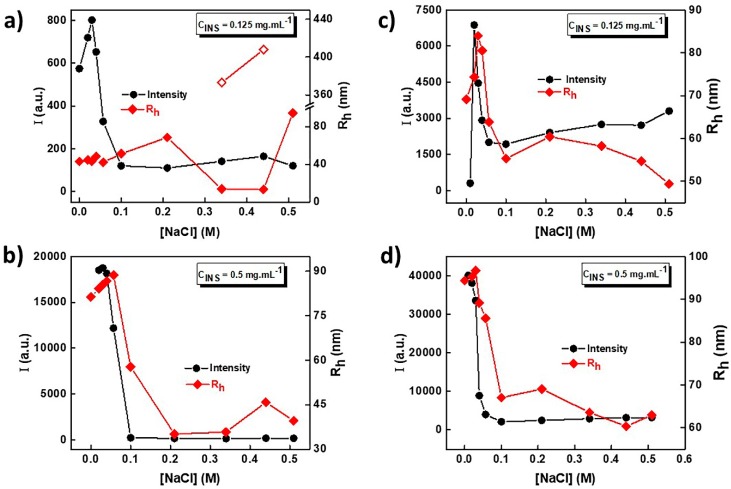
Scattering intensity and R_h_ as a function of ionic strength for QDPMAEMA_33_-*b*-PLMA_16_-*b*-POEGMA_30_/INS (**a**,**b**) and QPDMAEMA_13_-*b*-PLMA_39_-*b*-POEGMA_8_/INS complexes (**c**,**d**).

Figure 3 shows the variations in the scattering intensity and hydrodynamic radius, as a function of ionic strength, for the QPDMAEMA_33_-*b*-PLMA_16_-*b*-POEGMA_30_/INS (a, b) and QPDMAEMA_13_-*b*-PLMA_39_-*b*-POEGMA_8_/INS (b, d) complexes for C_INS_ = 0.125 mg·mL^−1^ and C_INS_ = 0.5 mg·mL^−1^.

**Scheme 3:**
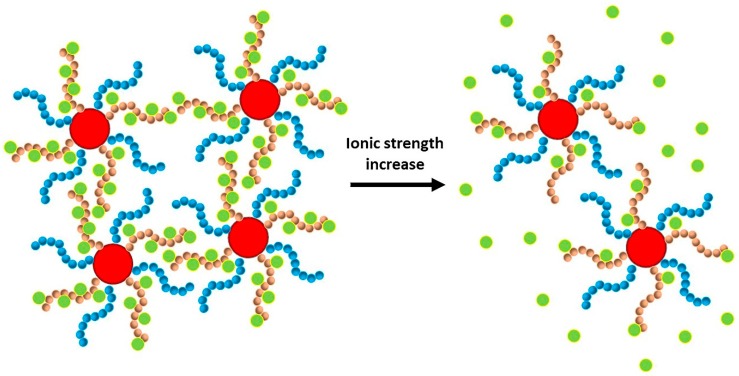
Effect of ionic strength on QPDMAEMA-*b*-PLMA-*b*-POEGMA/INS complexes.

For QPDMAEMA_33_-*b*-PLMA_16_-*b*-POEGMA_30_/INS complexes (C_INS_ = 0.125 mg·mL^−1^, Figure 3a) a small increase is observed at lower salt concentrations (C_NaCl_ = 0.01–0.03 M) and a rapid decrease is evident thereafter (C_NaCl_ = 0.03–0.1 M). This decrease can be translated as a decrease in the mass of the complexes, which eventually leads to their decomposition, showing that the complexes are not stable in the presence of salt. Α plateau can be observed at salt concentrations above C_NaCl_ = 0.1 M, where the scattering intensity remains practically constant. On the other side, the hydrodynamic radius does not change till C_NaCl_ = 0.1 M, then it increases and at higher salt concentrations two populations can be observed. One with larger size (350–400 nm, empty diamonds line in Figure 3a) and one with very small size (approx. 15 nm), but proportionally larger in scattering intensity. This fact shows the decomposition of the complexes and clusters of complexes, as salt concentration increases in the solution, since the small size population can be associated with the presence of free insulin while the larger population probably is associated with the presence of swollen clusters not entirely decomposed yet (see Scheme 3 for a graphical representation of the dissociation process).

For QPDMAEMA_33_-*b*-PLMA_16_-*b*-POEGMA_30_/INS complexes (C_INS_ = 0.5 mg·mL^−1^, Figure 3b), the scattering intensity does not change in lower salt concentrations (till C_NaCl_ = 0.06 M), it decreases rapidly from C_NaCl_ = 0.06 to 0.1 M and remains practically the same at higher salt concentrations. R_h_ follows the same pattern and after the initial small increase at lower salt concentrations, it decreases rapidly, showing the disorganization of the complexes.

However, in comparison with QPDMAEMA_33_-*b*-PLMA_16_-*b*-POEGMA_30_/INS complexes with C_INS_ = 0.125 mg·mL^−1^, this occurs at somehow lower salt concentrations, because the higher concentration of insulin may render the complexes less stable, probably due to the more loose structures formed in that case.

The scattering intensity and hydrodynamic radius of QPDMAEMA_13_-*b*-PLMA_39_-*b*-POEGMA_8_/INS complexes as a function of ionic strength, present similar behavior with the one discussed above at the corresponding insulin concentrations. Therefore, as far as the solution ionic strength effects on the structure and stability are concerned these are more pronounced and distinct for the two terpolymer/insulin systems at lower concentrations of insulin. 

**Figure 4:**
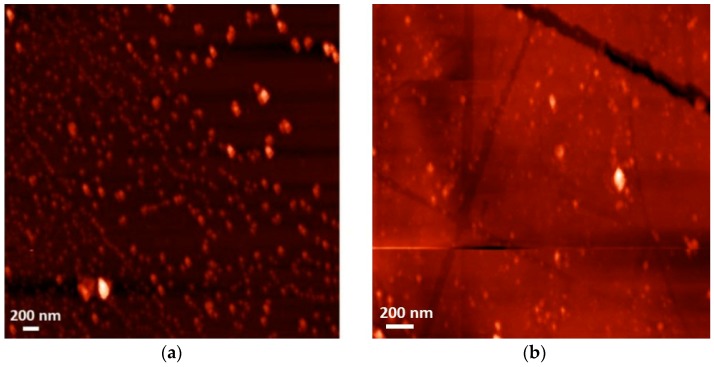
AFM images of (**a**) QPDMAEMA_33_-*b*-PLMA_16_-*b*-POEGMA_30_/INS complexes (C_INS_ = 0.5 mg·mL^−1^), (**b**) QPDMAEMA_13_-*b*-PLMA_39_-*b*-POEGMA_8_/INS complexes (C_INS_ = 0.5 mg·mL^−1^).

AFM measurements were performed to have a more complete picture about the morphology of QPDMAEMA-*b*-PLMA-*b*-POEGMA/INS complexes. Indicative AFM images are presented in Figure 4. The existence of both isolated particles (primary micelle/protein complexes) and aggregates (clusters of primary complexes) with average size in the range of 130–150 nm (diameter) is observed, as well as some larger aggregates formed by coalescence of the species on the mica surface. The height of the particles has been found to be around 100 nm, smaller than the dimensions at xy level, meaning that there is interaction with the substrate (mica) or that the structure of the complexes is rather loose and collapse of the structures occurs after their deposition on the substrate and the solvent removal. Such a loose structure is expected for polyelectrolyte/protein complexes due to their hydrophilic character and their ability to trap water in their interior. Even in the case of terpolymer micelles the larger part of the polymeric component is taken up by the swollen hydrophilic mixed QPDMAEMA/POEGMA corona compared to the space occupied by the hydrophobic PLMA cores.

Spectra from FS measurements at excitation wavelength 280 nm are going to give information about the conformation of the complexed protein, through the intrinsic fluorescence of the hydrophobic amino acid tyrosine (Tyr) that exists in both insulin chains.

**Figure 5:**
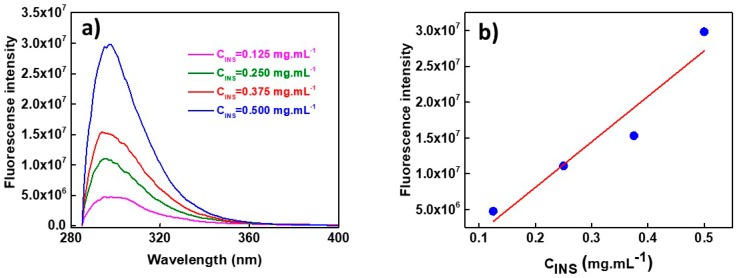
(**a**) Fluorescence spectra for QPDMAEMA_13_-*b*-PLMA_39_-*b*-POEGMA_8_/INS complexes, (**b**) Fluorescence intensity at 300 nm (peak maximum) as a function of INS concentration.

The fluorescence spectra of QPDMAEMA_13_-*b*-PLMA_39_-*b*-POEGMA_8_/INS at all insulin concentrations are presented in Figure 5a and the fluorescence intensity at peak maximum graphs as a function of INS concentration at 300 nm (peak maximum wavelength) are presented in Figure 5b. The deviation of the wavelength in which the maximum protein fluorescence intensity is observed is less than 10 nm, showing that there are no significant changes in the conformation of the protein after the formation of terpolymer/INS complexes. It is obvious that the fluorescence intensity increases as INS concentration in the solution increases in a rather linear fashion showing no precipitation in the solutions of the complexes (and in some way the stability of the complexes as insulin concentration increases). 

**Figure 6:**
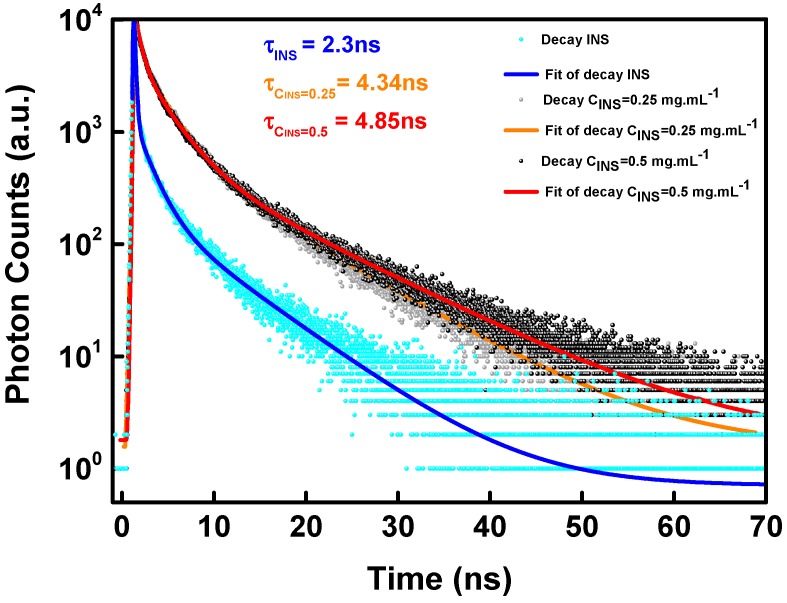
Photoluminescence lifetime decay graphs for INS (blue), QPDMAEMA13-*b*-PLMA39-*b*-POEGMA8/INS (C_INS_ = 0.25 mg·mL^−1^, grey) and QPDMAEMA13-*b*-PLMA39-*b*-POEGMA8/INS (C_INS_ = 0.5 mg·mL^−1^, black). Excitation wavelength was 280 nm and the emission probed at 300 nm.

Time-resolved FS measurements were performed on QPDMAEMA-*b*-PLMA-*b*-POEGMA/INS complexes to investigate the events that take place during the lifetime of the excited singlet state of the intrinsic tyrosine fluorescence. The results are presented in Figure 6. An increase in the relaxation time (average values shown in Figure 6) is observed as INS concentration in the solution increases. The increase is dramatic if compared with the relaxation time for free INS. This observation can be attributed to a more stereochemically constrained environment for tyrosine, and subsequently for significant crowding of the whole protein molecules participating in the complexes, because of the strong complexation with the cationic QPDMAEMA chains and their localization within the palisade of the micellar corona.

## 4. Conclusions

The ability of QPDMAEMA-*b*-PLMA-*b*-POEGMA cationic triblock terpolymer micelles to form complexes with insulin was demonstrated in this work. The positively charged micelles interact electrostatically with the negatively charged insulin forming complexes and clusters of complexes.

All the QPDMAEMA-*b*-PLMA-*b*-POEGMA/INS complexes solutions that were colloidally stable at all insulin concentrations used. The size of the complexes/clusters was found to increase as the protein concentration in the solution increased and the positive values of ζ-potential shows that the addition of insulin leads to formation of aggregates with a large number of positively charged segments in the periphery. This observation is also supported from AFM measurements, showing the existence of aggregates and single particles (complexes) in the solution. FS measurements show that the conformation of the protein is not affected after the complexation with the polymeric micelles and that the protein experiences a largely constrained environment within the complexes/clusters. Thus, the terpolymers can be used further for insulin delivery applications, due also to the small size of QPDMAEMA-*b*-PLMA-*b*-POEGMA/INS complexes formed.

## Figures and Tables

**Table 1:** Molecular characteristics of QPDMAEMA-*b*-PLMA-*b*-POEGMA triblock terpolymers.

Sample	M_w_^a^ (×10^4^) (g·mol^−1^)	M_w_/M_n_^a^	%wt. QPDMAEMA ^b^	%wt. PLMA ^b^	%wt. POEGMA ^b^
QPDMAEMA_33_-*b*-PLMA_16_-*b*-POEGMA_30_	2.83	1.46	35	14	51
QPDMAEMA_13_-*b*-PLMA_39_-*b*-POEGMA_8_	1.75	1.19	22	57	21

^a^ Determined by size exclusion chromatography (SEC), ^b^ Determined by proton nuclear magnetic resonance (^1^H-NMR).

## References

[1] Cho N.H., Shaw J.E., Karuranga S., Huang Y., da Rocha Fernandes J.D., Ohlrogge A.W., Malanda B. (2018). IDF Diabetes Atlas: Global estimates of diabetes prevalence for 2017 and projections for 2045. Diabetes Res. Clin. Pract..

[2] Mansoor S., Kondiah P.P.D., Choonara Y.E., Pillay V. (2019). Polymer-Based Nanoparticle Strategies for Insulin Delivery. Polymers.

[3] Vecchio I., Tornali C., Bragazzi N.L., Martini M. (2018). The Discovery of Insulin: An Important Milestone in the History of Medicine. Front. Endocrinol..

[4] Shah R.B., Patel M., Maahs D.M., Shah V.N. (2016). Insulin delivery methods: Past, present and future. Int. J. Pharm. Investig..

[5] Feng H., Lu X., Wang W., Kang N.-G., Mays J.W. (2017). Block Copolymers: Synthesis, Self-Assembly, and Applications. Polymers.

[6] Eliyahu H., Barenholz Y., Domb A.J. (2005). Polymers for DNA delivery. Molecules.

[7] Adams M.L., Lavasanifar A., Kwon G.S. (2003). Amphiphilic block copolymers for drug delivery. J. Pharm. Sci..

[8] Gohy J.-F., Abetz V. (2005). Block Copolymer Micelles. Block Copolymers II.

[9] Mintzer M.A., Simanek E.E. (2009). Nonviral Vectors for Gene Delivery. Chem. Rev..

[10] Lee K.Y., Yuk S.H. (2007). Polymeric protein delivery systems. Prog. Polym. Sci..

[11] Zhao H., Lin Z.Y., Yildirimer L., Dhinakar A., Zhao X., Wu J. (2016). Polymer-based nanoparticles for protein delivery: Design, strategies and applications. J. Mater. Chem. B.

[12] Tan Z., Jiang Y., Ganewatta M.S., Kumar R., Keith A., Twaroski K., Pengo T., Tolar J., Lodge T.P., Reineke T.M. (2019). Block Polymer Micelles Enable CRISPR/Cas9 Ribonucleoprotein Delivery: Physicochemical Properties Affect Packaging Mechanisms and Gene Editing Efficiency. Macromolecules.

[13] Dobrynin A.V., Rubinstein M. (2005). Theory of polyelectrolytes in solutions and at surfaces. Prog. Polym. Sci..

[14] Becker A.L., Henzler K., Welsch N., Ballauff M., Borisov O. (2012). Proteins and polyelectrolytes: A charged relationship. Curr. Opin. Colloid Interface Sci..

[15] Alshamsan A., Haddadi A., Incani V., Samuel J., Lavasanifar A., Uludağ H. (2009). Formulation and Delivery of siRNA by Oleic Acid and Stearic Acid Modified Polyethylenimine. Mol. Pharm..

[16] Sun T.-M., Du J.-Z., Yan L.-F., Mao H.-Q., Wang J. (2008). Self-assembled biodegradable micellar nanoparticles of amphiphilic and cationic block copolymer for siRNA delivery. Biomaterials.

[17] Pack D.W., Hoffman A.S., Pun S., Stayton P.S. (2005). Design and development of polymers for gene delivery. Nat. Rev. Drug Dis..

[18] Luo Y.Y., Xiong X.Y., Tian Y., Li Z.L., Gong Y.C., Li Y.P. (2016). A review of biodegradable polymeric systems for oral insulin delivery. Drug Deliv..

[19] Samal S.K., Dash M., Van Vlierberghe S., Kaplan D.L., Chiellini E., van Blitterswijk C., Moroni L., Dubruel P. (2012). Cationic polymers and their therapeutic potential. Chem. Soc. Rev..

[20] Kamenova K., Haladjova E., Grancharov G., Kyulavska M., Tzankova V., Aluani D., Yoncheva K., Pispas S., Petrov P. (2018). Co-assembly of block copolymers as a tool for developing novel micellar carriers of insulin for controlled drug delivery. Eur. Polym. J..

[21] Xie J., Li A., Li J. (2017). Advances in pH-Sensitive Polymers for Smart Insulin Delivery. Macromol. Rapid Commun..

[22] Pippa N., Kalinova R., Dimitrov I., Pispas S., Demetzos C. (2015). Insulin/poly(ethylene glycol)-block-poly(L-lysine) Complexes: Physicochemical Properties and Protein Encapsulation. J. Phys. Chem. B.

[23] Kumari A., Yadav S.K., Yadav S.C. (2010). Biodegradable polymeric nanoparticles based drug delivery systems. Colloids Surf. B.

[24] Al-Tahami K., Oak M., Mandke R., Singh J. (2011). Basal level insulin delivery: In vitro release, stability, biocompatibility, and in vivo absorption from thermosensitive triblock copolymers. J. Pharm. Sci..

[25] Skandalis A., Pispas S. (2017). PDMAEMA-b-PLMA-b-POEGMA triblock terpolymers via RAFT polymerization and their self-assembly in aqueous solutions. Polym. Chem..

[26] Karayianni M., Pispas S., Chryssikos G.D., Gionis V., Giatrellis S., Nounesis G. (2011). Complexation of Lysozyme with Poly(sodium(sulfamate-carboxylate)isoprene). Biomacromolecules.

[27] Quinn R., Andrade J.D. (1983). Minimizing the aggregation of neutral insulin solutions. J. Pharm. Sci..

[28] Lee A.S., Bütün V., Vamvakaki M., Armes S.P., Pople J.A., Gast A.P. (2002). Structure of pH-Dependent Block Copolymer Micelles: Charge and Ionic Strength Dependence. Macromolecules.

